# Analysis of Factors Affecting Postoperative Opioid Requirement in Pediatric Patients Undergoing Pectus Excavatum Repair with Multimodal Analgesic Management

**DOI:** 10.3390/jcm12165240

**Published:** 2023-08-11

**Authors:** Jung Min Koo, Hyung Joo Park, Gong Min Rim, Kwanyong Hyun, Jaewon Huh, Hoon Choi, Yunji Kim, Wonjung Hwang

**Affiliations:** 1 Department of Anesthesiology and Pain Medicine, Seoul St. Mary’s Hospital, College of Medicine, The Catholic University of Korea, Seoul 02706, Republic of Korea; miniyaa623@gmail.com (J.M.K.); ether@catholic.ac.kr (J.H.); hoonie83@naver.com (H.C.); 2 Department of Thoracic and Cardiovascular Surgery, Nanoori Hospitals, Seoul 06048, Republic of Korea; hyjpark@catholic.ac.kr (H.J.P.); yim8585@hanmail.net (G.M.R.); 3 Department of Thoracic and Cardiovascular Surgery, St. Vincent’s Hospital, College of Medicine, The Catholic University of Korea, Suwon 16247, Republic of Korea; k.hyun@catholic.ac.kr; 4 Department of Anesthesiology and Pain Medicine, Uijeongbu St. Mary’s Hospital, College of Medicine, The Catholic University of Korea, Bucheon-si 11765, Republic of Korea; sudasurunyunji1@gmail.com

**Keywords:** multimodal analgesia, pain, postoperative, pain management, pectus excavatum, pediatrics

## Abstract

Children with pectus excavatum are treated with surgical repair in a procedure known as minimally invasive repair of pectus excavatum (MIRPE). MIRPE causes considerable postoperative pain, resulting in the administration of a substantial dose of opioids. This study aimed to identify perioperative factors that influence the requirement for opioids in children undergoing MIRPE. Retrospective data from children who underwent MIRPE were analyzed. A multimodal analgesic protocol was implemented with a continuous wound infiltration system and administration of non-opioid analgesics. Intravenous opioid analgesics were administered if the pain score was greater than 4. The cumulative opioid use was assessed by calculating the morphine equivalent dose at 6, 24, and 48 h after surgery. Perioperative factors affecting the postoperative opioid use were identified with multiple linear regression analyses. This study included 527 children aged 3–6 years, with a mean age of 3.9 years. Symmetrically depressed chest walls, a lower Haller index, and a lower revised depression index were found to be associated with decreased postoperative opioids. Boys required higher opioid doses than girls. Longer pectus bars (10 inches versus 9 inches) were associated with increased opioid use. Severity indices, gender, and the length of pectus bars influence postoperative opioid requirement in children undergoing MIRPE surgery with multimodal analgesia.

## 1. Introduction

Pectus excavatum is the most common congenital deformity of the chest wall and affects more males than females [1,2]. Surgical repair is the only treatment to correct cardiopulmonary compression and cosmetic problems caused by the deformity. While repair of pectus excavatum is mainly performed in adolescents with an average age of about 15 years, children over 3 years of age are also tolerable to minimally invasive pectus excavatum repair (MIRPE), with minimal risk for complications such as pneumothorax, bar dislocation, and need of reoperation [3]. Surgical correction at a young age has the advantage of preventing chest wall asymmetry, growth retardation, and psychological distress.

MIRPE involves minimal incisions and does not require the rib cartilages to be cut, as in the traditional surgeries. Nevertheless, it is associated with severe postoperative pain lasting for several days [1,4,5]. Optimal pain control is essential to improve the postoperative recovery and prognosis in pediatric patients [6,7]. Inadequate pain control in children can lead to long-lasting pain memory, behavioral disorders, and chronic postsurgical pain. Similarly, a significant number of children who undergo MIRPE continue to suffer from both physiological and psychological distress months after the surgery, which is associated with a higher level of immediate postoperative pain [8]. However, pediatric pain is often inadequately controlled because of difficulties faced by children to accurately express pain and by professionals to evaluate pain. Thus, prediction of postoperative pain and analgesic requirement is necessary when planning the appropriate pain control regimen in young patients.

In this study, we investigated the perioperative factors that affect postoperative opioid requirement in preschoolers undergoing MIRPE with multimodal analgesia.

## 2. Materials and Methods

### 2.1. Study Population

We collected data from preschoolers aged 3–6 years who underwent pectus excavatum repair using pectus bars in a single university hospital between August 2012 and March 2019. This study was approved by the institutional review board (KC19RESI0271) and has been conducted in adherence with the STROBE guidelines. The data were retrospectively collected from the electronic medical records of patients. Patients were excluded if they had an American Society of Anesthesiologists score of class III or greater, history of pectus excavatum repair, history of chronic pain or preoperative analgesic medications, or inability to accurately express pain due to psychiatric or other disorders such as mental retardation or cerebral palsy. Patients with insufficient or missing data on clinical variables were also excluded from data analysis.

### 2.2. Surgical Technique

A single surgeon exclusively performed the surgical procedures on all patients who were included in this study. The surgical technique utilized in each case was customized based on the individual patient’s unique anatomical features and the severity of the underlying disease. With the patient positioned supine, general anesthesia was administered to the patients. Once the routine draping was completed, both arms were gently suspended overhead using specialized arm slings to maintain an optimal surgical field. For accurate localization of the insertion point for the pectus bar, the surgeon carefully examined and identified the ideal placement by considering the depressed area and bilateral hinge points on the patient’s chest. The pectus bars were skillfully shaped by the surgeon directly at the operating table, meticulously considering the unique morphology of the patient’s chest wall. The appropriate size of the pectus bar was determined based on precise measurements of the horizontal length of the chest wall. Throughout the study, both nine- and ten-inch pectus bars were utilized depending on the patient’s specific needs. In cases where the chest wall presented long or wide depressed areas, the surgeon strategically employed multiple bars to achieve the desired surgical outcome.

A small midaxillary skin incision, measuring approximately 1 cm in length, was made on both sides of the chest to introduce the pectus bar. Utilizing pectoscopic visualization and a 20 Fr chest tube as a guide, the bar was carefully passed below the depressed sternum and guided across the mediastinum. To effectively elevate the previously depressed chest wall, the surgeon adeptly flipped the pectus bar by 180° using a specialized flipper. This technique successfully lifted the sternum into its proper position, restoring the normal anatomical alignment. To ensure stability and to prevent any unwanted mobilization of the pectus bar, the surgeon expertly secured it in place using either claw fixators or bridge plates, as deemed appropriate for each patient’s unique situation.

Following the fixation of the pectus bars, an immediate anterior–posterior view of the chest was obtained using portable radiography in the operating room. This crucial step allowed for real-time verification of the accurate positioning of the bars and served as an additional safety check to detect any potential complications, such as pneumothorax. In cases where no major bleeding occurred during the intraoperative period, the placement of drains was deemed unnecessary, further contributing to the patient’s comfort and postoperative recovery.

### 2.3. Anesthetic and Analgesic Management

According to the institutional protocol, anesthesia was induced with ketamine (1–2 mg/kg) and maintained with sevoflurane and supplemental fentanyl (1–2 mcg/kg) based on vital signs and bispectral index (BIS). Sevoflurane was maintained within 2.0–2.5%, with an air/oxygen mixture comprising 40 to 50% inspired oxygen concentration. The concentration of sevoflurane was adjusted to keep the BIS values between 40 and 60, ensuring optimal anesthesia depth throughout the surgical procedure.

Prior to tracheal intubation, the administration of 0.6 mg/kg of rocuronium facilitated smooth and controlled intubation. Mechanical ventilation of 4–6 mL/kg of tidal volume was continued until the end of the surgery. Upon completion of the surgery, neuromuscular blockade was reversed with 0.2 mg/kg of pyridostigmine and 0.1 mg/kg of glycopyrrolate. After tracheal extubation, patients were transferred to the recovery room, where they continued to receive close monitoring and care.

As per the multimodal analgesic protocol, a comprehensive approach was adopted, encompassing both intraoperative and postoperative analgesic measures. Following the insertion of the pectus bar, the thoracic surgeon administered intercostal blocks and implanted a continuous wound infiltration system (CWIS, ON-Q^®^ Pain relief system, Halyard, Alpharetta, GA, USA), providing a sustained release of local anesthetics to the affected area. The intercostal blocks involved the percutaneous administration of 0.5–0.75% ropivacaine mixed with 0.9% saline at the 4 to 9th intercostal levels. Each level received 2 mL of local anesthetics, carefully targeted to provide optimal pain relief and minimize postoperative discomfort. The CWIS employed a main pump that contained the local anesthetics. The pump was connected to infusion catheters, equipped with multiple holes to ensure an even distribution of the local anesthetics. The catheter was inserted through the bilateral posterior axillary lines to infuse the local anesthetic around the corresponding intercostal nerve territory. The local anesthetic was aseptically prepared by diluting 270 mL ropivacaine (0.15–0.25%) in normal saline. The concentration of ropivacaine varied based on the patient’s body weight, with 0.15% for those weighing less than 15 kg, 0.2% for those between 16 and 20 kg, and 0.25% for those between 21 and 25 kg. The diluted ropivacaine was continuously administered at a controlled rate of 4 mL/h, and the pump remained in place for up to 72 h postoperatively, ensuring a sustained and controlled release of the local anesthetic to manage pain effectively.

In the recovery unit, the Wong–Baker FACES pain scale, designed to assess pain in children as young as 3 years old, was employed to measure pain severity every 10 min. This scale, consisting of a series of facial expressions ranging from 0 (happy face without pain) to 10 (crying face with the worst pain a child can have), provided a reliable and standardized means of quantifying pain. In cases where the pain score exceeded 4, experienced healthcare providers promptly administered intravenous boluses of opioid analgesics to alleviate pain and ensure the patient’s comfort. The selection of opioids, including fentanyl, pethidine, or nalbuphine, was based on the anesthesiologist’s preference.

Multimodal analgesic protocol was conducted even after the patients were sent to the ward. The fundamental oral form of analgesic medication was ibuprofen. A dosage of 100–150 mg oral ibuprofen was administered three or four times a day. Experienced healthcare providers assessed the severity of pain every 4 h using the Wong–Baker FACES pain scale. If the pain score was greater than 4, intravenous analgesics were administered. These analgesics was administered in the order of fentanyl (1 mcg/kg)–pethidine (1 mg/kg)–ketorolac (0.4–1 mg/kg). The intravenous analgesics were used in addition to the oral form to ensure rapid and immediate pain relief, particularly during the postoperative fasting period when oral administration might not be feasible.

### 2.4. Severity Index and Opioid Requirement

We analyzed the Haller and depression indices from the patients’ CT scans to measure the severity of pectus excavatum. The Haller index (HI) was calculated by dividing the transverse chest diameter by the anterior–posterior diameter from the posterior surface of the sternum to the anterior surface of the vertebral body [9]. The depression index (DI) was derived from the absolute measurement of sternal depression divided by the transverse diameter of the vertebral body. We also measured the revised depression index (rDI), derived from the ratio of the vertical distances between the actual and proposed post-repair chest wall to the transverse chest diameter [10]. An author of this study has developed innovative techniques for repairing asymmetric and severe pectus excavatum. New CT indices, including rDI, have been introduced to address the diverse morphological variations of pectus excavatum and facilitate customized techniques tailored to each specific case.

The cumulative opioid requirement was calculated as morphine equivalent dose (MED)/kg at 6, 24, and 48 h postoperatively. Doses of opioids other than morphine were converted to morphine equivalents using standard equianalgesic dose conversion ratios (i.e., 10 mg morphine equals 100 mcg fentanyl and 10 mg morphine is equianalgesic with 100 mg of pethidine). Wong–Baker FACES pain scales were also recorded at the same time points.

### 2.5. Statistical Analysis

Data are presented as mean ± standard deviation or absolute values (percentage), as appropriate. The main outcome was postoperative cumulative opioid consumption. Multiple linear regression analyses were performed to explore the factors influencing the main outcome and to adjust for potential confounding factors. Independent variables selected for inclusion in the model were derived from previously published data and clinical or biological plausibility. These factors included age, sex, height, weight, chest wall symmetry, HI, DI, rDI, length and number of pectus bars used, number of claw fixators used, administered dose of intraoperative fentanyl, concentration of ropivacaine within the CWIS, and cumulative dose of non-opioid analgesics at each time point. SPSS Statistics 18.0 for Windows (IBM Corp., Armonk, NY, USA) was used for statistical analyses. *p*-values < 0.05 were considered to indicate statistical significance.

## 3. Results

### 3.1. Study Population and Intraoperative Variables

Data from 560 patients were retrospectively collected, and 33 patients were excluded due to missing data. Therefore, data from 527 patients were analyzed in this study. Table 1 presents the demographic data of study participants. This study included 406 boys (77.0%) and 121 girls (23.0%), with a mean age of 3.9 ± 0.9 years. The average values of HI, DI, and rDI were 4.5, 0.7, and 1.8, respectively. Symmetrical cases were more prevalent than asymmetrical cases, comprising 83.5% of the total study population. Table 2 shows intraoperative variables of study participants. The average surgical duration was approximately 70 min. Cases involving the use of one bar and six fixators were more common than cases with two bars and more fixators, and the utilization of 9-inch bars was more frequent than that of 10-inch bars. Ropivacaine was administered through the CWIS at a concentration of 0.15%, 0.2%, and 0.25% in 395, 106, and 26 patients, respectively. The administered dose of fentanyl during surgery was 1.5 ± 1.1 mcg/kg.

### 3.2. Postoperative Variables

Table 3 provides a comprehensive overview of the postoperative pain scores and analgesic utilization. Among the patients in the recovery room, fentanyl emerged as the most-administered opioid (*n* = 353, 67%), followed by nalbuphine (*n* = 21) and pethidine (*n* = 10). A considerable proportion of patients (*n* = 143, 27%) did not require any opioids in the recovery room, indicating that the intraoperative multimodal analgesic protocol sufficed for pain management without the need for rescue analgesics. Over the postoperative period, pain scores exhibited a significant decline by 24 h following the surgery. Moreover, the necessity for opioid analgesics steadily diminished from 6 to 24 and 48 h postoperatively. The incremental changes in cumulative morphine equivalent dose gradually decreased during this time course. Conversely, the demand for non-opioid analgesics (ketorolac) remained relatively constant at each time point, as mentioned above.

In Table 4, we present a summary of the results obtained from multiple linear regression analyses conducted at 6, 24, and 48 h postoperatively. The factors influencing postoperative opioid usage at each time point are presented in descending order of their significance. Remarkably, the symmetry of chest walls, HI, and rDI emerged as significant factors influencing postoperative opioid requirement. Patients with asymmetric chest walls, higher HI values, and higher rDI values were correlated with increased opioid consumption after surgery. Female gender was identified as the sole factor that negatively impacted opioid consumption. Girls required lower opioid doses compared to boys across all time points. At 6 h after the end of surgery, the length of the pectus bar exhibited the highest correlation with opioid consumption, followed by female gender and the asymmetry of the chest wall. Notably, patients who received a 10-inch bar necessitated greater opioid doses than those who received a 9-inch bar. Additionally, the length of the pectus bar and the presence of an asymmetric chest wall demonstrated positive correlations with increased opioid consumption from 6 to 24 h postoperatively. Subsequently, the rDI and HI showed positive correlations with opioid consumption from 24 to 48 h postoperatively.

Intriguingly, other preoperative factors, such as age, height, weight, and DI, did not significantly influence postoperative opioid use. Moreover, the number of bars or claw fixators employed did not appear to affect opioid consumption after surgery.

## 4. Discussion

We identified perioperative factors that affect postoperative opioid requirements in preschoolers undergoing MIRPE. To the best of our knowledge, this is the first study to investigate postoperative pain following MIRPE in children aged 3–6 years. We analyzed the records of more than 500 patients during an 8-year period, creating one of largest pediatric datasets to date. Our results demonstrate that symmetric chest wall, lower HI and rDI, female sex, and shorter bar length were associated with less postoperative opioid requirement.

The results of this study were similar to those of an earlier study that reported that the severity and asymmetry of pectus excavatum affect postoperative opioid analgesic use in patients with a median age of 17 years [11]. The study found a 6% increase in opioid requirement for every 1 cm increase in depression depth, defined as the distance between the deepest point and the expected location of the patient’s sternum after correction. Another study also demonstrated a correlation between the severity of pectus excavatum and the force required to elevate the depressed sternum to the desired level [12]. If greater force is applied during correction, there is a higher likelihood of increased tissue damage. Hence, it is plausible that the rDI, reflecting the anticipated degree of correction, may significantly correlate with opioid requirement after surgery.

Additionally, the length of the bar was associated with the opioid use during the acute postoperative period. The presence of the bar in the chest can generate postoperative pain due to constant pressure on the ribs and tension applied to the bar’s extremities [13]. We postulate that a longer pectus bar would induce greater external pressure and muscle retraction, leading to greater tissue injury and subsequently an increased demand for opioids in the postoperative period. The length of the bar is determined by the patient’s chest wall distance, but selecting a shorter length of the bar whenever possible would be helpful in reducing postoperative opioid consumption.

In the present study, girls required fewer opioids compared to boys, which contrasts with previous studies in preschool children and adolescents [14,15]. In a study involving pectus excavatum repair in adults, women reported higher pain intensity and greater opioid requirement after repair compared to men [16]. However, another study found that women used 23 to 43% fewer opioid doses after various types of elective surgery compared to men [17]. These conflicting results are due to significant variations in the extent of surgery or the age of the subjects in each study. As of yet, no established conclusion exists for a similar age group and surgical range to those in this study.

Understanding the sex difference in pain intensity and opioid requirement is challenging because pain is a multifactorial phenomenon. Proposed hypotheses include variances in endogenous opioids, neurotransmitters, post-pubertal hormonal effects, and culture-specific gender roles [18,19]. Unlike adults, hormonal factors are unlikely to significantly impact opioid use in preschool children. A possible explanation for higher opioid requirements in boys is their vigorous movements and activities after surgery. Further studies are needed to confirm sex differences in pain perception and opioid consumption among children undergoing various surgical procedures.

Adequate postoperative pain control is crucial for enhancing recovery and improving prognosis [6,20]. While opioids have traditionally played a significant role in postoperative pain management, their use is associated with perioperative complications. For this reason, a multimodal analgesic approach, which includes regional anesthesia, local anesthetics, and non-opioid agents, has been recommended. This approach is now widely implemented for children undergoing MIRPE, as it is considered more ideal than relying solely on a single modality [21,22]. In the following study, regional anesthetic management including intercostal blocks and application of a wound infiltration system provided sufficient analgesia to 143 (27%) children in the immediate postoperative period in the recovery unit. We postulate that these children had residual analgesic effects from the regional block and wound infiltration of local anesthetics.

At our institution, we have implemented multimodal analgesia consisting of CWIS and regular administration of non-opioid analgesics for over 10 years in approximately 900 pediatric patients. CWIS was chosen due to its comparable analgesic effect, ease of application, shorter surgery time, and reduced hospital stay compared to thoracic epidural block [23]. Our protocol has provided effective analgesia, leading to a 60% reduction in postoperative opioid consumption. As a result, we observed a 40% decrease in the incidence of postoperative nausea/vomiting and 70% decrease in constipation associated with opioid use. Only a small number of patients (1–2 per year) required early removal of CWISs due to catheter displacement or kinking, and no other significant complications such as catheter-related infections were observed. Based on our experience, we believe that the use of CWISs is a reliable and safe component of multimodal analgesia in children undergoing MIRPE.

Assessing pain in pediatric patients after surgery poses unique challenges due to their limited ability to communicate their feelings and discomfort verbally. This is particularly true for very young children, such as those aged 3 to 4 years, who may not possess the verbal and cognitive skills to express their pain accurately. As a result, healthcare professionals employ various methods and pain assessment tools to evaluate and understand pain intensity in this population, aiming to ensure appropriate pain management and improve the overall well-being of these young patients.

The Wong–Baker FACES pain scale is a widely used pediatric pain assessment tool in both clinical and research settings. [24,25] This scale features a series of faces ranging from a smiling face on the left, representing “no hurt”, to a crying face on the right, indicating “hurts worst”. The scale typically comprises six faces, each depicting different levels of pain intensity. Dr. Wong and Baker compared different pain assessment scales in pediatrics [26]. Their findings showed that children prefer the faces scale over other scales and the faces pain scale is not inferior in terms of its validity or reliability. Among different facial expression scales, most of which demonstrate adequate to excellent psychometric properties, we have chosen the Wong–Baker FACES pain scale because it can be measured in children as young as 3 years old. The Wong–Baker FACES pain scale proves effective and efficient in assessing pain in children, especially in those who might have difficulty expressing their pain verbally or when language barriers are present.

Some of the key advantages of the Wong–Baker FACES pain scale are its ease of use, simplicity, and quick administration. In addition, the Wong–Baker FACES pain scale demonstrated relatively high reliability and validity in postoperative assessments. It exhibits high correlations with other validated pain assessment methods, such as visual analog scales, thereby establishing its reliability and consistency in measuring pain intensity.

This study had several limitations. First, it was a retrospective study with some missing variables. Second, the Wong–Baker FACES pain rating scale includes subjective measurements. Although it is a common pain measurement tool in children, it can be inaccurate if the evaluator is inexperienced. However, we consider our data to be reliable because the scale was administered by professional healthcare providers. They are well trained and experienced since they work in a ward where the majority of the patients are children who have received MIRPE. Therefore, our findings may be of value in planning pain management in young children undergoing surgery of a similar extent.

## 5. Conclusions

In conclusion, asymmetric chest wall, higher Haller index and revised depression index, males, and the insertion of longer chest bars increase postoperative opioid use in pediatric patients undergoing MIPRE. The anesthetic and surgical management of these patients based on our findings may be helpful for adequate postoperative analgesia.

## Figures and Tables

**Table 1:** Patient demographics.

Gender	
Males	406 (77.0)
Females	121 (23.0)
Age (years)	3.9 ± 0.9
Height (cm)	105 ± 7.9
Weight (kg)	17.5 ± 6.2
Haller index	4.5 ± 1.5
Depression index	0.7 ± 0.4
Revised depression index	1.8 ± 0.5
Symmetry of depression	
Symmetric	440 (83.5)
Asymmetric	87 (16.5)

Values are expressed as numbers (proportion) or mean ± standard deviation.

**Table 2:** Intraoperative variables.

Operation time (min)	69.9 ± 20.9
Number of bars inserted	
1	514 (97.5)
2	13 (2.5)
Length of bar inserted (inch)	
9	372 (70.6)
10	155 (29.4)
Number of fixators inserted	
6	512 (97.2)
<6	15 (2.8)

Values are expressed as numbers (proportion) or mean ± standard deviation.

**Table 3:** Postoperative variables.

Time Points	6 h	24 h	48 h
Pain score	4.4 ± 1.0	1.9 ± 0.5	1.8 ± 0.6
Opioid			
Frequency	512 (97.2)	191 (36.2)	149 (28.3)
Dose (MED ^a^/kg)	0.26 ± 0.14	0.06 ± 0.08	0.03 ± 0.06
Cumulative dose (MED ^a^/kg)	0.26 ± 0.14	0.32 ± 0.16	0.35 ± 0.17
Non-opioid (Ketorolac)			
Frequency	124 (23.5)	115 (21.8)	100 (19.0)
Dose (mg/kg)	0.2 ± 0.4	0.2 ± 0.4	0.2 ± 0.4

Values are expressed as number (proportion) or mean ± standard deviation. ^a^ MED: morphine equivalent dose.

**Table 4:** Factors affecting accumulative opioid consumption at (a) 6 h, (b) 24 h, and (c) 48 h after surgery.

(a)						
Dependent Variable	Independent Variables	B	β	t	*p* Value	VIF
MED ^a^/kg at 6 h	(Factor)	0.922				
	Length of pectus bar (≥10 inches)	0.554	0.187	4.195	<0.001	1.049
	Female	−0.389	−0.122	−2.787	0.006	1.036
	Asymmetry	0.335	0.092	2.104	0.036	1.023
	R = 0.668 R^2^ = 0.446 adj R^2^ = 0.441 D-W = 1.053 F = 91.809 *p* < 0.001					
(**b**)						
**Dependent Variable**	**Independent Variables**	**B**	**β**	**t**	***p* Value**	**VIF**
MED ^a^/kg at 24 h	(Factor)	1.503				
	Revised depression index	0.704	0.185	2.857	0.004	1.020
	Length of chest bar (≥10 inches)	0.708	0.178	4.077	<0.001	1.036
	Female	−0.635	−0.148	−3.379	0.001	1.036
	Haller index	0.150	0.131	2.024	0.004	1.021
	Asymmetry	0.525	0.108	2.468	0.014	1.019
	R = 0.676 R^2^ = 0.457 adj R^2^ = 0.453 D-W = 1.333 F = 120.423 *p* < 0.001					
(**c**)						
**Dependent Variable**	**Independent Variables**	**B**	**β**	**t**	***p* Value**	**VIF**
MED ^a^/kg at 48 h	(Factor)	2.787				
	Revised depression index	1.095	0.208	3.194	0.001	1.020
	Female	−1.094	−0.184	−4.203	<0.001	1.019
	Haller index	−0.256	0.162	2.490	0.013	1.036
	R = 0.656 R^2^ = 0.431 adj R^2^ = 0.426 D-W = 1.481 F = 86.795 *p* < 0.001					

^a^ MED: morphine equivalent dose.

## Data Availability

The data generated in this study can be shared after a reasonable request to the corresponding author.

## References

[1] Muhly W.T., Beltran R.J., Bielsky A., Bryskin R.B., Chinn C., Choudhry D.K., Cucchiaro G., Fernandez A., Glover C.D., Haile D.T. (2019). Perioperative Management and In-Hospital Outcomes after Minimally Invasive Repair of Pectus Excavatum: A Multicenter Registry Report From the Society for Pediatric Anesthesia Improvement Network. Anesth. Analg..

[2] De Oliveira Carvalho P.E., da Silva M.V., Rodrigues O.R., Cataneo A.J. (2014). Surgical interventions for treating pectus excavatum. Cochrane Database Syst. Rev..

[3] Park H.J., Sung S.W., Park J.K., Kim J.J., Jeon H.W., Wang Y.P. (2012). How early can we repair pectus excavatum: The earlier the better?. Eur. J. Cardiothorac. Surg..

[4] Papic J.C., Finnell S.M., Howenstein A.M., Breckler F., Leys C.M. (2014). Postoperative opioid analgesic use after Nuss versus Ravitch pectus excavatum repair. J. Pediatr. Surg..

[5] Fonkalsrud E.W., Beanes S., Hebra A., Adamson W., Tagge E. (2002). Comparison of minimally invasive and modified Ravitch pectus excavatum repair. J. Pediatr. Surg..

[6] Ferland C.E., Vega E., Ingelmo P.M. (2018). Acute pain management in children: Challenges and recent improvements. Curr. Opin. Anesthesiol..

[7] Walker S.M. (2015). Pain after surgery in children: Clinical recommendations. Curr. Opin. Anesthesiol..

[8] Uhl K.M., Wilder R.T., Fernandez A., Huang H., Muhly W.T., Zurakowski D., Cravero J.P. (2020). Postoperative pain and psychological outcomes following minimally invasive pectus excavatum repair: A report from the Society for Pediatric Anesthesia Improvement Network. Paediatr. Anaesth..

[9] Sesia S.B., Heitzelmann M., Schaedelin S., Magerkurth O., Kocher G.J., Schmid R.A., Haecker F.M. (2019). Standardized Haller and Asymmetry Index Combined for a More Accurate Assessment of Pectus Excavatum. Ann. Thorac. Surg..

[10] Park H. (2011). Minimally Invasive Surgery for Pectus Excavatum: Park Technique. J. Clin. Anal. Med..

[11] Grosen K., Pfeiffer-Jensen M., Pilegaard H.K. (2010). Postoperative consumption of opioid analgesics following correction of pectus excavatum is influenced by pectus severity: A single-centre study of 236 patients undergoing minimally invasive correction of pectus excavatum. Eur. J. Cardiothorac. Surg..

[12] Fonkalsrud E.W., Reemtsen B. (2002). Force required to elevate the sternum of pectus excavatum patients. J. Am. Coll. Surg..

[13] Ghionzoli M., Ciuti G., Ricotti L., Tocchioni F., Lo Piccolo R., Menciassi A., Messineo A. (2014). Is a shorter bar an effective solution to avoid bar dislocation in a Nuss procedure?. Ann. Thorac. Surg..

[14] Karišik M., Gligorović Barhanović N., Vulović T., Simić D. (2019). Postoperative pain and stress response: Does child’s gender have an influence?. Acta Clin. Croat..

[15] Nafiu O.O., Thompson A., Chiravuri S.D., Cloyd B., Reynolds P.I. (2019). Factors Associated With Recovery Room Intravenous Opiate Requirement after Pediatric Outpatient Operations. Anesth. Analg..

[16] Ravanbakhsh S., Farina J.M., Bostoros P., Abdelrazek A., Mi L., Lim E., Mead-Harvey C., Arsanjani R., Peterson M., Gotimukul A. (2022). Sex Differences in Objective Measures of Adult Patients Presenting for Pectus Excavatum Repair. Ann. Thorac. Surg..

[17] Chia Y.Y., Chow L.H., Hung C.C., Liu K., Ger L.P., Wang P.N. (2002). Gender and pain upon movement are associated with the requirements for postoperative patient-controlled iv analgesia: A prospective survey of 2298 Chinese patients. Can. J. Anaesth..

[18] Orhan C., Van Looveren E., Cagnie B., Mukhtar N.B., Lenoir D., Meeus M. (2018). Are Pain Beliefs, Cognitions, and Behaviors Influenced by Race, Ethnicity, and Culture in Patients with Chronic Musculoskeletal Pain: A Systematic Review. Pain. Physician.

[19] Bartley E.J., Fillingim R.B. (2013). Sex differences in pain: A brief review of clinical and experimental findings. Br. J. Anaesth..

[20] Boric K., Dosenovic S., Jelicic Kadic A., Batinic M., Cavar M., Urlic M., Markovina N., Puljak L. (2017). Interventions for postoperative pain in children: An overview of systematic reviews. Paediatr. Anaesth..

[21] Archer V., Robinson T., Kattail D., Fitzgerald P., Walton J.M. (2020). Postoperative pain control following minimally invasive correction of pectus excavatum in pediatric patients: A systematic review. J. Pediatr. Surg..

[22] Mavi J., Moore D.L. (2014). Anesthesia and analgesia for pectus excavatum surgery. Anesthesiol. Clin..

[23] Choudhry D.K., Brenn B.R., Sacks K., Reichard K. (2016). Continuous chest wall ropivacaine infusion for analgesia in children undergoing Nuss procedure: A comparison with thoracic epidural. Paediatr. Anaesth..

[24] Tomlinson D., von Baeyer C.L., Stinson J.N., Sung L. (2010). A systematic review of faces scales for the self-report of pain intensity in children. Pediatrics.

[25] Stinson J.N., Kavanagh T., Yamada J., Gill N., Stevens B. (2006). Systematic review of the psychometric properties, interpretability and feasibility of self-report pain intensity measures for use in clinical trials in children and adolescents. Pain.

[26] Wong D.L., Baker C.M. (1988). Pain in children: Comparison of assessment scales. Pediatr. Nurs..

